# A 5′-tRNA halve, tiRNA-Gly promotes cell proliferation and migration via binding to RBM17 and inducing alternative splicing in papillary thyroid cancer

**DOI:** 10.1186/s13046-021-02024-3

**Published:** 2021-07-05

**Authors:** Litao Han, Hejing Lai, Yichen Yang, Jiaqian Hu, Zhe Li, Ben Ma, Weibo Xu, Wanlin Liu, Wenjun Wei, Duanshu Li, Yu Wang, Qiwei Zhai, Qinghai Ji, Tian Liao

**Affiliations:** 1 Department of Head and Neck Surgery, Fudan University Shanghai Cancer Center, Fudan University, Shanghai, 200032 China; 2 Department of Oncology, Shanghai Medical College, Fudan University, Shanghai, 200032 China; 3 CAS Key Laboratory of Nutrition, Metabolism and Food Safety, CAS Center for Excellence in Molecular Cell Sciences, Shanghai Institute of Nutrition and Health, University of Chinese Academy of Sciences, Chinese Academy of Sciences, Shanghai, 200031 China; 4 School of Life Science and Technology, Shanghai Tech University, Shanghai, 200093 China; 5 Fudan University Shanghai Cancer Center, Key Laboratory of Medical Epigenetics and Metabolism, Institutes of Biomedical Sciences, Fudan University, Shanghai, 200032 China

**Keywords:** tiRNA-Gly, RBM17, Alternative splicing, MAP4K4, Papillary thyroid carcinoma

## Abstract

**Background:**

tRNA-derived small noncoding RNAs (sncRNAs) are mainly categorized into tRNA halves (tiRNAs) and fragments (tRFs). Biological functions of tiRNAs in human solid tumor are attracting more and more attention, but researches concerning the mechanisms in tiRNAs-mediated tumorigenesis are rarely. The direct regulatory relationship between tiRNAs and splicing-related proteins remain elusive.

**Methods:**

Papillary thyroid carcinoma (PTC) associated tRNA fragments were screened by tRNA fragments deep sequencing and validated by qRT-PCR and Northern Blot in PTC tissues. The biological function of tRNA fragments were assessed by cell counting kit, transwells and subcutaneous transplantation tumor of nude mice. For mechanistic study, tRNA fragments pull-down, RNA immunoprecipitation, Western Blot, Immunofluorescence, Immunohistochemical staining were performed.

**Results:**

Herein, we have identified a 33 nt tiRNA-Gly significantly increases in papillary thyroid cancer (PTC) based on tRFs & tiRNAs sequencing. The ectopic expression of tiRNA-Gly promotes cell proliferation and migration, whereas down-regulation of tiRNA-Gly exhibits reverse effects. Mechanistic investigations reveal tiRNA-Gly directly bind the UHM domain of a splicing-related RNA-binding protein RBM17. The interaction with tiRNA-Gly could translocate RBM17 from cytoplasm into nucleus. In addition, tiRNA-Gly increases RBM17 protein expression via inhibiting its degradation in a ubiquitin/proteasome-dependent way. Moreover, RBM17 level in tiRNA-Gly high-expressing human PTC tissues is upregulated. In vivo mouse model shows that suppression of tiRNA-Gly decreases RBM17 expression. Importantly, tiRNA-Gly can induce exon 16 splicing of *MAP4K4* mRNA leading to phosphorylation of downstream signaling pathway, which is RBM17 dependent.

**Conclusions:**

Our study firstly illustrates tiRNA-Gly can directly bind to RBM17 and display oncogenic effect via RBM17-mediated alternative splicing. This fully novel model broadens our understanding of molecular mechanism in which tRNA fragment in tumor cells directly bind RNA binding protein and play a role in alternative splicing.

**Supplementary Information:**

The online version contains supplementary material available at 10.1186/s13046-021-02024-3.

## Background

Small RNA fragments from tRNAs are a kind of sncRNAs derived from tRNAs or the precursors of tRNAs [1, 2]. Before and after exportation to the cytoplasm, pre-tRNAs and mature tRNAs undergo extensive cleavage, resulting in the production of two main types of tRNA-derived sncRNAs: tRNA halves (tiRNAs) and tRNA-derived fragments (tRFs) [3]. tiRNAs, consisting of 30–35 nt 5′-tRNA halves and 40–50 nt 3′-tRNA halves, were generated from cleavage of the anticodon loops by ribonuclease angiogenin [1, 2]. In the last decade, accumulating evidences found that tiRNAs were associated with tumor process. Honda et al. firstly unveiled a new tiRNA, sex hormone-dependent tRNA-derived RNAs (SHOT-RNAs), could enhance cell proliferation in breast and prostate cancers, which was hormone and receptor-dependent [4]. These evidences have showed that tiRNAs may be involved in tumorigenesis in hormone-dependent cancers. Subsequently, several 5′-tiRNAs expression patterns have been identified in clear cell renal cell carcinoma. They were inversely correlated with tumor stage and grade [5, 6]. Recent researches began to show that tiRNAs play a vital role on cancer trait including cell proliferation, cell cycle and cancer metastasis in certain tumors [7–9], but the functional implication of tiRNAs in those tumors remains to be clarified. Lately, 5′-tiRNA-Val has been proved to inhibit Wnt/β-catenin signaling pathway through targeting FZD3 mRNA 3′-UTR in breast cancer cells [10]. Roughly, few findings have showed that the aberrant expressed tiRNAs in human solid tumors could be promising diagnostic biomarkers or therapeutic targets. However, whether tiRNAs could directly combine with proteins and further regulate the function of its protein partners in tiRNAs-mediated tumorigenesis are largely unknown.

In this study, we aimed to illustrate the role and underlying mechanism of tiRNAs involved in the carcinogenesis of papillary thyroid carcinoma (PTC). Using the tRFs & tiRNAs sequencing analysis, we identified five tRFs & tiRNAs which were differentially expressed in PTC tissue. The expression level of tiRNA-Gly was the highest and increasing tiRNA-Gly positively correlated with advanced pathologic grade and lymph nodes metastasis. Attentionally, we for the first time demonstrated that tiRNA-Gly could directly bind to an RNA-binding protein, RNA binding motif protein 17 (RBM17). RBM17 is a protein that can bind to spliceosome, participates in the alternative splicing of mRNAs [11]. Several studies have reported that RBM17 plays a crucial role in malignant tumor [12, 13]. It can interact with lncRNA Saf to induce exclusion and alternative splicing of *Fas* gene [14]. However, the regulatory mechanism of RBM17 in tiRNAs-mediated tumorigenesis are largely unknown. Our findings revealed that tiRNA-Gly can directly bind to RBM17 and promote the malignant activities of cancer cells via RBM17-mediated alternative splicing, indicating that tiRNA-Gly is extremely important in tumorigenesis through interacting with RBM17 and regulating alternative splicing of target genes.

## Methods

### Tissue samples

Ninety-one pairs of human PTC tissue samples and adjacent tissue samples were collected in the Department of Head and Neck Surgery at the Fudan University Shanghai Cancer Center between 2014 and 2018. All patients were pathologically confirmed to have papillary thyroid cancer, and no patients received prior treatment for their thyroid condition before surgery. Clinical data (sex, age, tumor size, extrathyroidal invasion, metastasis, multifocality and TNM classification) were obtained according to the 4th edition of the WHO Classification of Tumors of Endocrine Organs and the 8th edition of the AJCC/TNM staging system of thyroid cancer. All the patients provided written informed consent. This study was approved by the Ethics Committee of the Fudan University Shanghai Cancer Center.

### Cell culture

Three PTC cell lines (K1, TPC-1 and BCPAP) and the Nthy-ori 3–1 normal human thyroid cell line were used in this study. K1 and TPC-1 cells were purchased from the University of Colorado Cancer Center Cell Bank. BCPAP cells were purchased from the Typical Culture Preservation Commission Cell Bank, Chinese Academy of Sciences (Shanghai, China). Nthy-ori 3–1 cells were purchased from Sigma-Aldrich, Inc. All four cell lines were cultured in RPMI 1640 medium with 10% fetal bovine serum at 37 °C with 5% CO_2_ in proper humidity.

### tiRNAs&tRFs sequences processing

To identify tiRNAs&tRFs in papillary thyroid cancer, total RNAs were extracted from three pairs of T3-T4 stage (AJCC 8th edition) papillary thyroid cancer tissue samples using TRIzol reagent (Invitrogen, Carlsbad, California, USA), as the method mentioned previously [10]. Briefly, total RNAs was treated by 3′-aminoacyl deacylation, 5′-OH phosphorylation, and m1A and m3C demethylation. For tiRNAs&tRFs sequencing, 15 ~ 40 nt sncRNA biotypes were sequenced with an Illumina NextSeq 500 system. tRF and tiRNA data were analyzed by the Arraystar tRF&tiRNA-seq data package.

### RNA-seq and exon detection

RNA was extracted from K1 cells transfected with tiRNA-Gly or SCR with the above mentioned method. RNA samples were then sent for RNA-seq to detect alternative splicing. A 5′ primer (5′-CGAGCCCAAAGCCCACTA) and a 3′ primer (TCCTGCCTGGCTATTCTGC-3′) were designed to detect exon 15 and exon 17 of the MAP4K4 gene.

### Northern blotting

RNA samples were denatured at 90 °C for 3 min and chilled on ice immediately. After loading, the samples were separated on a 15% urea–TBE denaturing polyacrylamide gel and electrophoretically transferred to a nylon membrane (Roche, INYC00010, Mannheim, Germany). Following UV crosslinking for 8 min and prehybridization at 45 °C for 1 h, RNA was hybridized with a 3′-digoxigenin-labeled DNA probe (CAGTGGTAGAATTCTC-3′) at 45 °C overnight. The membrane was then washed, blocked for 3 h, and incubated with anti-digoxigenin antibody solution. The complexes were washed with digoxigenin washing buffer, after which CSPD substrate (Invitrogen, T2142, Carlsbad, CA, USA) was applied, and the membrane was placed on a development folder. A phosphor imaging screen was used to expose the membrane to X-ray film.

### RNA isolation and quantitative RT-PCR

To detect tiRNAs, total RNA (5 μg) was first demethylated using the ALKB enzyme. After treatment with T4 polynucleotide kinase (NEB, M0201L, USA) to dephosphorylate the 3′ ends, total RNA was then treated with poly(A) polymerase (NEB, M0276L, USA) to add adenosine monophosphate to the 3′ ends of the RNAs as tails. The total RNA was then used for first-strand cDNA synthesis with PrimeScript™ II 1st Strand cDNA Synthesis Kit (Takara, 6210A, Dalian, China). A poly(T) DNA primer containing a specific PCR primer (GCGAGCACAGAATTAATACGACTCACTATAGG(T)12VN) was designed and used to detect specific tiRNAs by quantitative RT-PCR (Takara, Dalian, China) using the SYBR Premix Dimer Eraser kit (Takara, RR091A, Dalian, China). For tiRNA-Gly detection, the PCR primers were listed below: 5′-TGGTAGAATTCTCGCCTAAAA, 3′-GCGAGCACAGAATTAATACGACTCACTATAGG. For total tRNA-Gly detection, the PCR primers were listed below: 5′-GCATTGGTGGTTCAGTGGTAGA, 3′-GCGAGCACAGAATTAATACGACTCACTATAGG. For RBM17 expression, the PCR primers were listed below: RBM17, 5′-AAGAGCGTTTCTTCTCCGTCT, 3′-CCACCTGCATTCTCCGATCA. U6 and β-actin were used as endogenous control for quantitative RT-PCR. The reactions were performed with an Applied Biosystems QuantStudio (TM) 7 Flex System. Fold changes in expression were calculated using the 2-^ΔΔ^Ct method.

### RNA transfection and siRNA design

tiRNA with a 5′ phosphate group (5′-P-tiRNA-Gly, 5′-P-GCAUUGGUGGUUCAGUGGUAGAAUUCUCGCCU-3′) were synthesized (GenScript, Nanjing, China) and transfected for 6 h using Lipofectamine 2000 (Invitrogen, Carlsbad, CA, USA). A scrambled sequence was used as a negative control.

siRNAs against tiRNA-Gly were designed to include the 5 nt before the 5′ end of the mature form of tiRNA-Gly; these siRNAs targeted only one isoform for interference and had the best cleavage site. The two siRNAs against tiRNA-Gly were as follows: siRNA-1, 5′-CAGUAGCAUUGGUGGUUCA-3′; and siRNA-2, 5′-AGUAGCAUUGGUGGUUCAG-3′. siRNAs against RBM17 were as follows: siRNA-1, 5′-GCAACTCCTTCCTCGCTAA-3′; siRNA-2, 5′-CCACTTCTCTGGTAGAGAA-3′; and siRNA-3, 5′-GGACTCAAGACCTCGATCA-3′.

### RNA pulldown assay

An RNA pulldown assay for tiRNA-binding proteins was modified based on a previous method [15]. In brief, biotin-labeled tiRNA-Gly (sense) and antisense were synthesized by GenScript. The RNA bait sequences were heated to 99 °C and placed on ice immediately to eliminate secondary structure. 50 μl of streptavidin Dynabeads (Invitrogen, 65,305, Carlsbad, CA, USA) was washed with binding buffer three times. The Dynabeads were resuspended in 200 μl of binding buffer and then mixed with sense and antisense RNA or a blank control. Then, the mixtures were incubated on a shaker for 4 h at 4 °C. 2 mg of protein lysate from TPC-1 cells was added to the tubes, which were rotated overnight at 4 °C. After washing with PBS three times on a magnetic separation rack, RNA-protein complexes were resuspended in PBS and denatured with SDS-PAGE loading buffer at 99 °C for 10 min. After electrophoresis on a 7.5% PAGE gel, proteins were developed with a Fast Silver Stain Kit (Beyotime, P0017S, Shanghai, China). Protein bands with significant differences were cut out and sent for protein mass spectrometry analysis.

### RNA immunoprecipitation (RIP) assay

K1 cells transfected with the pCMV-FLAG-RBM17 plasmid were subjected to RIP with EZ-Magna RIP™ RNA-Binding Protein Immunoprecipitation Kit (Millipore, 17–701, Bedford, MA, USA). In brief, cells were lysed with RIP lysis buffer containing protease inhibitor cocktail and RNase inhibitor. 50 μl of protein A/G magnetic beads was washed and mixed with 5 μg of rabbit anti-DYKDDDDK Tag antibody (Cell signaling technology, 14,793). After slow rotation for 4 h at 4 °C, the magnetic beads bound to antibody were separated and incubated with cell lysates overnight at 4 °C. The antibody-protein-RNA complexes were separated a magnetic stand. RNA was extracted and qualified by qRT-PCR as described before.

### Immunoprecipitations (IP)

K1 cells transfected with tiRNA-Gly were lysed with cell lysis buffer containing protease inhibitor cocktail. 50 μl of protein A/G magnetic beads was washed and mixed with 10 μg of rabbit anti-RBM17 antibody (Abcam, ab114968, Shanghai, China). After slow rotation for 4 h at 4 °C, the magnetic beads bound to antibody were separated and incubated with cell lysates overnight at 4 °C. The antibody-protein complexes were separated with a magnetic stand. Protein was extracted and qualified by Western Blotting.

### Subcellular fractionation assay

K1 cells were transfected with SCR or tiRNA-Gly as previously described. We used the standard protocol for NE-PER Nuclear and Cytoplasmic Extraction Reagents (Thermo Fisher, 78,833, Rockford, USA). In short, cells were scraped off a dish and collected by centrifugation at 1500×g for 5 min. After washing with PBS, 200 μl of CER I was added to the pellet for resuspension. Then, 11 μl of CER II was added, and the tube was centrifuged at 16000×g for 5 min. The supernatant consisted of cytosolic extract. The precipitate was lysed with 100 μl of NER buffer by vortexing. After 40 min of incubation on ice, the tube was centrifuged at 16000×g for 10 min. The supernatant consisted of nuclear extract. RNA and protein extracted from cytosolic extract and nuclear extract were subjected to RT-PCR and western blotting, respectively.

### Western blotting

Cells were collected and lysed with RIPA buffer (Yeasen, 20115ES60, Shanghai, China) supplemented with protease inhibitor (Roche, P8340, Shanghai, China) and phosphatase inhibitor (Roche, P0044, Mannheim, Germany). The wells of a 10% Tris-Bis gel were loaded with equal amounts of protein. After electrophoresis for 2 h, proteins were transferred to a PVDF membrane (Roche, 3,010,040,001, Bedford, MA, USA) in a Mini Trans-Blot. After incubation with target and secondary antibodies, the membrane was subjected to chemiluminescence (Millipore, WBKLS0050, Bedford, MA, USA). Antibodies against the following were used: rabbit anti-RBM17 antibody (Abcam, ab114968), rabbit anti-ubiquitin antibody (Abcam, ab7780). Rabbit anti-β-actin antibody (Cell signaling technology, 4970) and rabbit anti-GAPDH antibody (Cell signaling technology, 5174) were used as endogenous controls.

### Cell counting kit-8 (CCK-8) assay

Cell viability was measured by CCK-8 kit (Yeasen, 40203ES60, Shanghai, China). After transfection for 48 h, cells were plated in 96-well plates at a density of approximately 5 × 10^3^ cells per well. At fixed intervals, 10 μl of CCK-8 solution and 100 μl of complete culture medium were added to the wells after the original medium had been discarded. OD values (450 nm) were detected. All assays were repeated three times.

### Cell migration assay

Falcon cell culture inserts were placed in the wells of 12-well plates to which 500 μl of complete culture medium had been added. 200 μl of RPMI 1640 medium without FBS mixed with 1 × 10^5^ cells were loaded into the upper chamber. After incubation for 24 h, the upper chamber was wiped with a cotton swab so that cells that had not moved through the membrane were removed. The bottom of the filter was fixed with 4% paraformaldehyde in 0.1 M PBS and stained with a 0.5% crystal violet Staining solution (Beyotime, C0121, Shanghai, China). Photographs were taken in 5 random fields by microscopy. To ascertain the real effect on cell migration, we calculated the migrating cells after excluded the proliferating cells. All the transwell assay were corrected for the effect of proliferating cells.

### Immunofluorescence

K1 cells were seeded on glass-bottom culture dishes (Nest, 801,001). Twenty-four hours after transfection of tiRNA-Gly, cells were fixed in 4% paraformaldehyde in 0.1 M PBS, permeabilized with 0.5% Triton and incubated with rabbit anti-RBM17 antibody (Abcam, ab114968) overnight at 4 °C. After extensive washing, cells were stained with Alexa Fluor 594 AffiniPure Goat Anti-Rabbit IgG antibody (Yeasen, 33112ES60, Shanghai, China) for 1 h in the dark. After rinsing with PBS-BT three times, the cells were coated with DAPI mounting solution (Yeasen, 36308ES11, Shanghai, China). Images were immediately collected by confocal laser scanning microscopy (Leica TCS SP5) under a 63× oil objective. U2 OS cells were used in parallel as a positive control, and cells transfected with scramble RNA (SCR) were used as a negative control.

### Immunohistochemistry (IHC)

Samples were fixed with paraformaldehyde and embedded in paraffin. Slices were deparaffinized in xylene and rehydrated in descending concentrations of ethanol. After being soaked into 3% H_2_O_2_ and blocked with 5% normal serum in 1× TBS, each sample was incubated with rabbit anti-RBM17 antibody (Abcam, ab114968) overnight at 4 °C. HRP-conjugated goat anti-rabbit IgG (Yeasen, 33101ES60, Shanghai, China) was used as a secondary antibody. Slices were stained with diaminobenzidine (DAB) for development (Dako, DAB substrate chromogen system) and hematoxylin and eosin (H&E). Images were obtained with an Olympus IX71 microscope. All results were confirmed by two experienced independent pathologists. Staining scores based on the proportion of stained cells (0, no staining; 1, ≤10%; 2, 10–50%; 3, ≥50%) and staining intensity (0, negative; 1, weak; 2, moderate; 3, strong) were calculated.

### In vivo mouse model

Balb/c female nude mice were subcutaneously injected with 8 × 10^6^ TPC-1 cells. Every group consisted of ten nude mice. Animal weight and tumor volume were measured every 4 days. After the mice were anesthetized by isoflurane inhalation, 2 nmol of si-tiRNA-Gly (5′-CAGUAGCAUUGGUGGUUCA-3′, GenScript, Nanjing, China) was injected into the tumors at multiple sites every 4 days, and NS was injected into the control group. Four weeks later, the mice were killed. Tumors were resected and subjected to immunohistochemistry. Tumor volume was calculated using the following formula: V = L × W^2^ × 0.5 (V, volume; L, length; W, width).

### Statistical analysis

tRF and tiRNA sequences were analyzed by R package (version 3.51). Data are presented as the mean ± SD. Raw data from experiments run in triplicate normalized by mean ^Δ^Ct were compared using the t-test (two-tailed). Differences for which *p* < 0.05 were considered statistically significant. Analysis of variance (ANOVA) was performed for comparisons between three or more groups.

## Results

### Expression of tRFs and tiRNAs in human PTC tissues

**Fig. 1:**
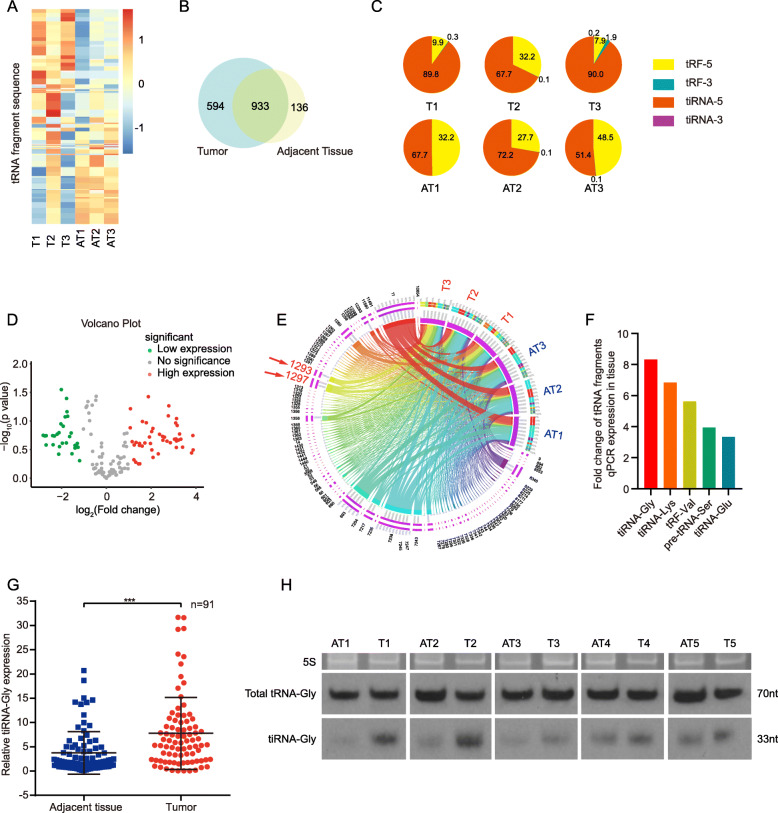
Expression of tRFs and tiRNAs in human PTC tissues. **A** Heatmap of tiRNAs and tRFs sequence in three pairs of tumors (T) and adjacent tissues (AT) of PTC. **B** 594 tRNA fragments were detected only in tumor samples (Blue region), 136 tRNA fragments only in adjacent tissues (Yellow region), and 933 tRNA fragments in both tissues (Green region). **C** 5′-tiRNA (tiRNA-5, orange part) and 5′-tRF (tRF-5, yellow part) account for the most two kinds of tRNA fragments. **D** Downregulated tRNA fragments (Green dots) and upregulated tRNA fragments (Red dots) in T compared to AT in volcano plot. Red dots means fold change > 2, Green dots means fold change < − 2. **E** Red arrows point out 5′-tiRNA-Gly-GCC (tiRNA-Gly). Upregulated and down regulated tiRNAs and tRFs in three pairs of T and AT tissue samples are displayed in circos plot. **F** Expression of selected 5 upregulated tRNA fragments was examined in 91 pairs of human PTC tissues by qRT-PCR. **G** tiRNA-Gly expression is higher in tumor tissues than in adjacent tissue in 91 pairs of human PTC by qRT-PCR. ***p < 0.001. **H** tiRNA-Gly is upregulated in tumor (T1–5) than adjacent tissues (AT1–5) by Northern blot (lower bands). Upper bands are 5S rRNA in denaturing gel. Middle bands are total tRNA-Gly

To identify tRFs and tiRNAs in papillary thyroid cancer tissues, three pairs of local advanced human PTC samples and adjacent tissues were collected for a high-throughput sequencing technique. The results were compared against the tRNA library GtRNAdb [16]. Mitochondrial tRNAs and their derivatives were excluded from the library. After normalization as previously described [17], 1723 tRNA fragments were identified in three pairs of tumor tissues (T) and their adjacent tissues (AT) (Fig. 1A); among these tRNA fragments, 594 tRNA fragments were detected in only tumor samples while 136 were detected in only ATs (Fig. 1B). Component analysis showed that tiRNAs in carcinoma particularly outnumbered tiRNAs in ATs, while tRFs seemed to be expressed at higher levels in ATs than in tumor tissues (Fig. 1C). A volcano plot showed that the following five tRNA fragments were present at significantly higher levels in tumor tissues than in ATs: 5′-tiRNA-Gly-GCC, 5′-tiRNA-Lys-CTT, 5′-tiRNA-Glu-CTC, 5′-tRF-Val-CAC, and pre-tRNA-Ser-TGA (Fig. 1D). As shown in Fig. 1E, tRNA fragments 1260, 1293, 1297, and 1358 were obviously derived from tumor tissues rather than ATs. tRNA fragments 1293 and 1297 were 5′-tiRNA-Gly-GCC spliced from different locations of tRNA-Gly-GCC. tRNA fragment 1260 had been cleaved from tRNA-Glu-CTC, and tRNA fragment 1358 had been cleaved from tRNA-Lys-CTT.

To confirm the results of tRFs&tiRNA-Seq data, we investigated the expression levels of above tRFs&tiRNAs in 91 pairs of PTC tissues and ATs by modified qRT-PCR (supplementary Fig. 1A). Among them, tiRNA-Gly had the highest expression level in human PTC tissues (Fig. 1F) and tiRNA-Gly was notably higher in tumor tissues than in ATs (*p* < 0.001) (Fig. 1G). For 63 of 91 (69.2%) pairs of tissues, tiRNA-Gly expression was higher in tumor tissues, while for 28 of 91 (30.8%) pairs of tissues, tiRNA-Gly expression was lower in tumor tissues than in ATs (supplementary Fig. 1B). tiRNA-Gly was cleaved from tRNA-Gly and the cleavage site was shown in supplementary Fig. 1C. Northern blot analysis showed that the tiRNA-Gly level in PTC tumor tissues was higher than that in ATs. Whereas, tRNA-Gly levels in tumor tissues and ATs were similar (Fig. 1H). We then analyzed the correlation between tiRNA-Gly expression and clinical characteristics of 91 PTC patients and found that elevated tiRNA-Gly expression was significantly positively correlated with tumor size, lymph node metastasis and TNM stage (Supplementary Fig. 1D and Supplementary Table 1). Altogether, we hypothesized that tiRNA-Gly may play an oncogenic role in the progression of PTC.

### tiRNA-Gly regulates cell proliferation and migration in PTC cells and influences tumor growth in mouse model

**Fig. 2:**
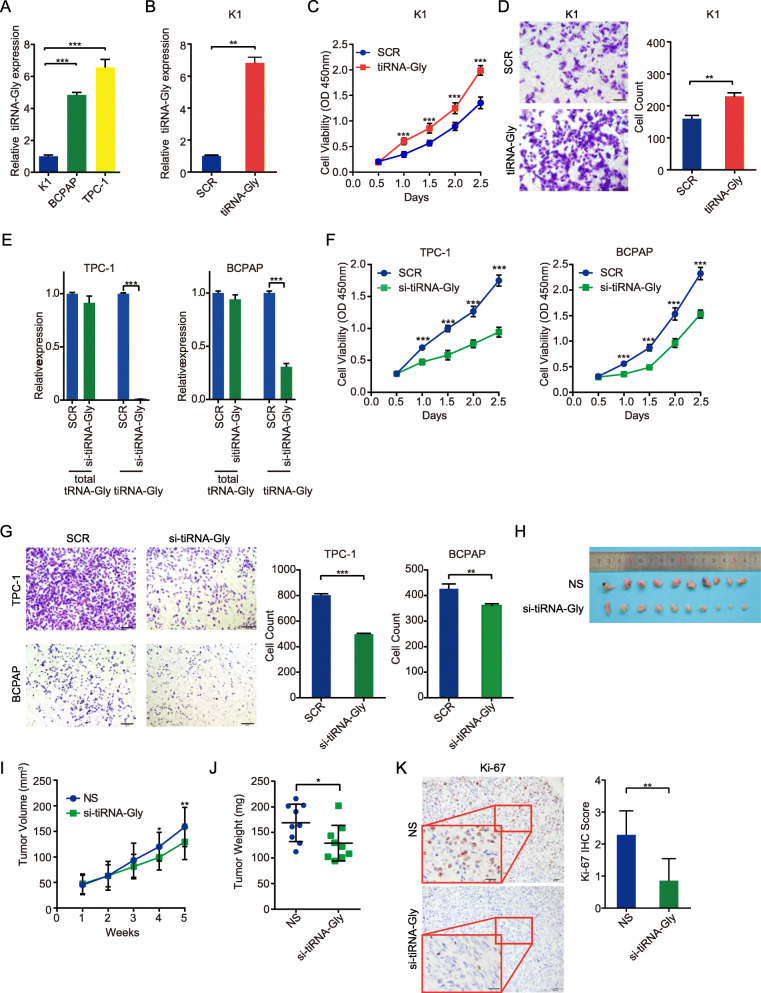
tiRNA-Gly regulates malignant activities of PTC cells in vitro and in vivo. **A** Relative expression of tiRNA-Gly in three human PTC cell lines detected by qRT-PCR. **B** Overexpression efficiency of tiRNA-Gly transfection in K1 cells by qRT-PCR. **C**-**D** tiRNA-Gly overexpression promotes K1 cells proliferation and migration by CCK-8 assay and migration assay compare to scramble sequence (SCR). Scale bar = 100 μm. **E** Knockdown efficiency of si-tiRNA-Gly on tiRNA-Gly and total tRNA-Gly in TPC-1 and BCPAP cells by qRT-PCR. **F**-**G** tiRNA-Gly knockdown suppresses TPC-1 and BCPAP cells proliferation and migration by CCK-8 assay and migration assay compare to scramble sequence (SCR). Scale bar = 100 μm. **H**-**J** Tumor growth in si-tiRNA-Gly group (*n* = 10) was suppressed in tumor volume and tumor weight compared with NS group (*n* = 10). **K** Ki-67 staining of tumors in si-tiRNA-Gly group is downregulated compared with NS group. Scale bar = 20 μm. Values are triple replicated and displayed in mean ± SD. *p < 0.05, **p < 0.01, ***p < 0.001

To study the biological role of tiRNA-Gly in PTC cells, we first examined tiRNA-Gly expression in three human PTC cell lines, K1, TPC-1 and BCPAP. As shown in Fig. 2A, K1 cells had far lower tiRNA-Gly levels than TPC-1 and BCPAP cells. For gain-of-function experiments, we synthesized tiRNA-Gly with a 5′ phosphate (5′-P-tiRNA-Gly) and transfected it into K1 cells. The transfection efficiency was tested by qRT-PCR (Fig. 2B). A cell viability assay demonstrated that tiRNA-Gly accelerated cell proliferation (Fig. 2C). A transwell assay revealed that tiRNA-Gly promoted the cell migration of K1 cells (Fig. 2D). For loss-of-function experiments, we transfected siRNA into TPC-1 and BCPAP cells to knock down tiRNA-Gly. The sequences of siRNAs designed to knock down tiRNA-Gly are shown in Supplementary Fig. 1E. qRT-PCR results pooled from ten times independent experiments showed the siRNA (si-tiRNA-Gly) significantly diminished tiRNA-Gly but have no significant influence on total tRNA-Gly in TPC-1 and BCPAP cells (Fig. 2E). As expected, si-tiRNA-Gly transfection inhibited the proliferation (Fig. 2F) and migration (Fig. 2G) of both TPC-1 and BCPAP cells. To see whether the reduction of total tRNA-Gly caused by si-tiRNA-Gly could affect cell viability, it needs to exclude the reduction of tiRNA-Gly caused by si-tiRNA-Gly. We designed si-tiRNA-Gly 2–4 (5′-CTGGAGCATTGGTGGTTCAG-3′), which can reduce the total tRNA-Gly about 15% in TPC-1 cells and 20% in BCPAP cells (Supplementary Fig. 1F) but have no effect on tiRNA-Gly (Supplementary Fig. 1G). Then, we transfected the si-tiRNA-Gly 2–4 into TPC-1 and BCPAP cells, and performed the CCK8 assay. The data showed that the reduction of total tRNA-Gly did not affect cell viability of both TPC-1 and BCPAP cells (Supplementary Fig. 1H). In short, the ectopic expression of tiRNA-Gly promoted the proliferation and migration of PTC cells, and vice versa tiRNA-Gly suppression inhibited the proliferation and migration of PTC cells.

To further confirm these results in vivo, we subcutaneously injected TPC-1 cells into nude mice. si-tiRNA-Gly was injected into the tumors every 4 days for a total of 5 injections, with normal saline (NS) used as a negative control. The knockdown effect of tiRNA-Gly in transplanted tumors of si-tiRNA-Gly group was shown in Supplementary Fig. 1I. Both tumor volume and tumor weight were markedly lower in si-tiRNA-Gly group than in NS group (Fig. 2H-J). Moreover, Ki-67 expression was also significantly lower in tumor tissues of si-tiRNA-Gly group compared to NS group (Fig. 2K). Our data demonstrated that knockdown of tiRNA-Gly decreased the tumor growth of PTC in vivo. Both in vitro and in vivo assays proved that tiRNA-Gly acted as an oncogenic factor in PTC.

### tiRNA-Gly directly interacts with RBM17 and regulates RBM17 expression in PTC cells

**Fig. 3:**
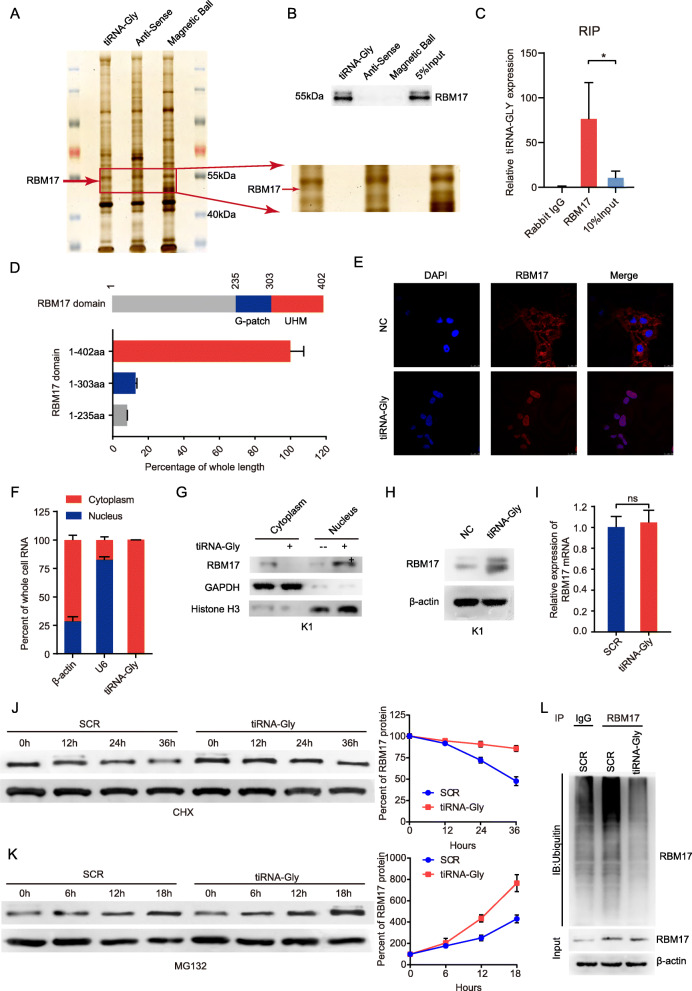
tiRNA-Gly directly binds to RBM17 and regulates RBM17 expression in PTC cells. **A** RNA pulldown assays are used to select proteins that interact with tiRNA-Gly in K1 cells. The red arrow marked the band where RBM17 located in tiRNA-Gly sense group in. Anti-sense and magnetic ball act as negative control. **B** The verification of RBM17 in RNA pulldown assays by western blot. 5% input acts as positive control. **C** RIP assays confirm the binding between endogenous RBM17 and tiRNA-Gly in K1 cells. Rabbit IgG acts as negative control, 10% input acts as positive control. Endogenous RBM17 antibody used in RIP assay. **D** RIP assays show deletion of the UHM (303–402 aa) of RBM17 significantly abolished the association between RBM17 full length (1–402 aa) and tiRNA-Gly. **E** Transfection tiRNA-Gly into K1 cells translocates RBM17 from cytoplasm into nucleus by immunofluorescence. Scale bar = 25 μm. **F** tiRNA-Gly is mainly located in cytoplasm by subcellular fractionation assay. β-actin served as the cytoplasmic internal control. U6 served as the nuclear internal control. Values are expressed as the mean ± SEM. **G** Transfection tiRNA-Gly into K1 cells decreases cytoplasmic RBM17 protein levels and increases nuclear RBM17 protein levels in K1 cells by western blot. GAPDH acts as cytoplasm control and Histone H3 as nucleus control. **H** and **I** Total RBM17 protein level is upregulated **H**) and total RBM17 mRNA level is not influenced **I**) after tiRNA-Gly is transfected into K1 cells. β-actin acts as the internal control. **J** K1 cells are treated with cycloheximide (CHX; 50 μg/ml) and transfected with tiRNA-Gly or scramble sequence (SCR) for indicated times. DMSO acts as a control group. Following treatment of CHX, the half-life of endogenous RBM17 protein is increased in tiRNA-Gly transfected K1 cells by Western blot. β-actin referred as a control. RBM17 qualification result shows in right. **K** K1 cells are treated with MG132 (20 μg/ml) and transfected with tiRNA-Gly or scramble sequence (SCR) for indicated times. DMSO acts as a control group. Following treatment with MG132, the accumulation of endogenous RBM17 in K1 cells transfected with tiRNA-Gly is largely higher compared to K1 cells transfected with scrambled siRNA by Western blot. β-actin referred as a control. RBM17 qualification result shows in right. **L** The binding of RBM17 and ubiquitinated protein is suppressed in K1 cells transfected with tiRNA-Gly by IP assay. Data are representative immunoblots of three independent assays. Values are triple replicated and displayed in mean ± SD. ***p < 0.001

To explore the molecular mechanism underlying the role of tiRNA-Gly in PTC carcinogenesis, we synthesized biotin-labeled tiRNA-Gly and its antisense sequence and performed RNA pulldown assays to select proteins that interact with tiRNA-Gly in K1 cells. The results from two independent tiRNA-Gly pulldown assays revealed a ~ 50 kDa band (Fig. 3A). Proteins with a peptide count > 3 and unique peptide count > 3 determined via mass spectrometry were selected. Five potential tiRNA-Gly-interacting proteins were obtained due to their presence in the band containing the sense sequence, but not that containing the antisense sequence, in two independent experiments (Supplementary Table 2). Among them, RBM17 was verified by three independent pulldown assays with the sense sequence, but not the antisense sequence (Fig. 3B). RNA immunoprecipitation (RIP) assays also proved that tiRNA-Gly was enriched when antibody against RBM17 was used for the pulldown assay (Endogenous RBM17 antibody used in RIP assay shown in Fig. 3C and anti-FLAG antibody used in RIP assay shown in Supplementary Fig. 2A). RNA immunoprecipitation (RIP) assays also proved that tiRNA-Gly was enriched when antibody against RBM17 was used for the pulldown assay (Fig. 3C). Further RIP assays using FLAG-tagged full-length and truncated RBM17 showed that deletion of the UHM (303–402 aa) of RBM17 (truncated RBM17) significantly abolished the association between RBM17 full length (1–402 aa) and tiRNA-Gly (Fig. 3D), indicating that tiRNA-Gly binds to the UHM of RBM17. Moreover, interaction with tiRNA-Gly facilitated the translocation of RBM17 from the cytoplasm into the nuclei of K1 cells (Fig. 3E), although tiRNA-Gly was mainly localized in the cytoplasm (Fig. 3F), what’s more, resulted in decreased cytoplasmic RBM17 protein levels and increased nuclear RBM17 protein levels in K1 cells (Fig. 3G and Supplementary Fig. 2B). Together, these results indicate that tiRNA-Gly binds to the RBM17 UHM and facilitates the translocation of RBM17 from the cytoplasm into the nucleus.

Given that tiRNA-Gly interacts with RBM17 and facilitates its translocation, we wanted to investigate the further molecular consequences of this interaction. Interestingly, tiRNA-Gly upregulated the total RBM17 protein level (Fig. 3H and Supplementary Fig. 2C) but did not influence the RBM17 mRNA level in K1 cells (Fig. 3I). Following treatment with the protein synthesis inhibitor cycloheximide (CHX; 50 μg/ml), tiRNA-Gly transfection increased the half-life of endogenous RBM17 protein in K1 cells, with β-actin as a relative control (Fig. 3J). Moreover, following treatment with the proteasome inhibitor MG132, the accumulation of endogenous RBM17 in K1 cells transfected with tiRNA-Gly was largely higher than that in K1 cells transfected with scrambled siRNA, suggesting that tiRNA-Gly inhibits the proteasome-dependent degradation of RBM17 in PTC cells (Fig. 3K). Furthermore, overexpression of tiRNA-Gly significantly suppressed the levels of ubiquitinated RBM17 (Fig. 3L). Collectively, these results demonstrated that tiRNA-Gly can stabilize the RBM17 protein through ubiquitin/proteasome-dependent degradation.

### RBM17 promotes the proliferation and migration of PTC cells and is elevated in PTC tumor tissues

**Fig. 4:**
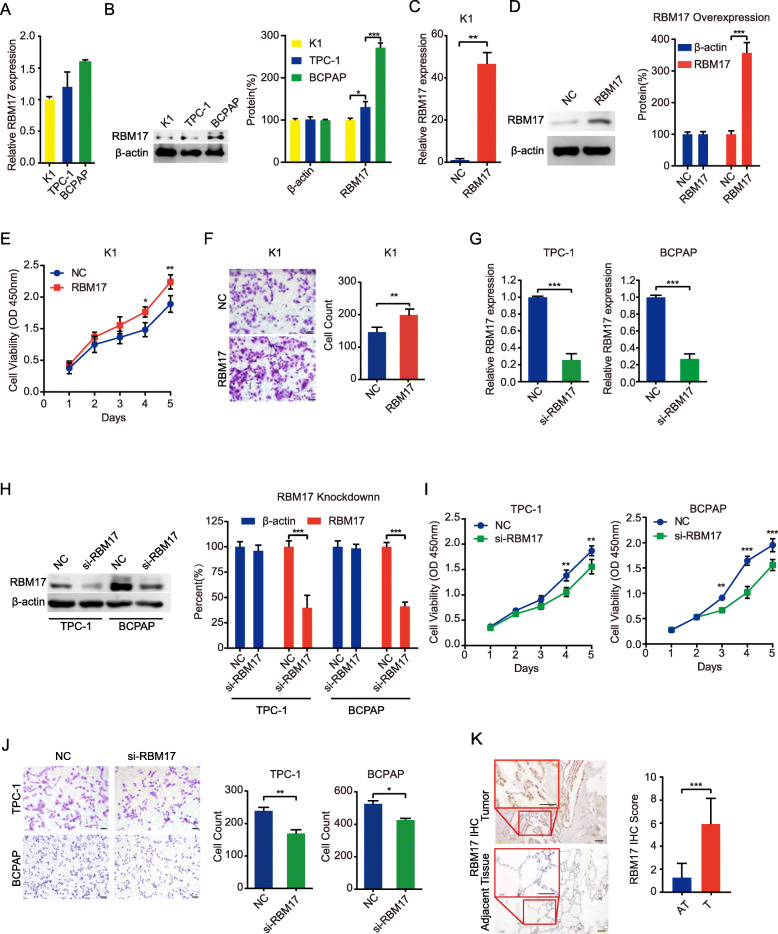
RBM17 promotes cell proliferation and migration of PTC cells. **A**-**B** RBM17 expression in three human PTC cell lines by qRT-PCR and western blot. β-actin acts as control. **C**-**D** Overexpression efficiency of RBM17 in K1 cell transfected with pCMV-RBM17 plasmid by qRT-PCR and Western blot with its quantification result. β-actin referred as a control. Negative control (NC) means control group. **E**-**F** RBM17 overexpression promotes proliferation and migration of K1 cells by CCK-8 assay and migration assay compare to vector group (NC). Scale bar = 100 μm. **G**-**H** Knockdown efficiency of RBM17 in TPC-1 and BCPAP cells transfected with siRNA-RBM17 by qRT-PCR and western blot with its quantification result. β-actin referred as a control. **I**-**J** RBM17 knockdown suppresses proliferation and migration of K1 cells by CCK-8 assay and migration assay compare to vector group (NC). Scale bar = 100 μm. **K** RBM17 expression in tumor (T) is higher than in adjacent tissues (AT) in 91 pairs PTC patients by IHC. Scale bar = 20 μm. Values are triple replicated and displayed in mean ± SD. *p < 0.05, **p < 0.01, ***p < 0.001

To investigate the function of RBM17 in PTC cells, we firstly detected its expression in the K1, TPC-1 and BCPAP PTC cell lines. Both the mRNA and protein levels of RBM17 were lower in K1 cells than in TPC-1 cells and BCPAP cells, similar to the trend in tiRNA-Gly expression in the three PTC cell lines (Fig. 4A-B). Then, we constructed the pCMV-RBM17 plasmid to overexpress RBM17 in K1 cells (Fig. 4C-D). Cell Counting Kit-8 (CCK-8) and migration assays showed that ectopic expression of RBM17 accelerated the proliferation and migration of K1 cells (Fig. 4E-F). RBM17 was knocked down in TPC-1 and BCPAP cells using siRNA transfection. The knockdown efficiency was confirmed by qRT-PCR and western blotting (Fig. 4G-H). Knockdown of RBM17 decreased the proliferation and migration of both TPC-1 and BCPAP cells (Fig. 4I-J). As shown in Fig. 4K, the RBM17 protein level in PTC tumor tissues was significantly increased compared to its level in ATs. These results indicate that RBM17 might be an oncogenic driver in the carcinogenesis of PTC, consistent with the effect of tiRNA-Gly on PTC.

### Attenuation of RBM17 rescues the effect of tiRNA-Gly on PTC cells and the expression of RBM17 in PTC tissues is regulated by tiRNA-Gly

**Fig. 5:**
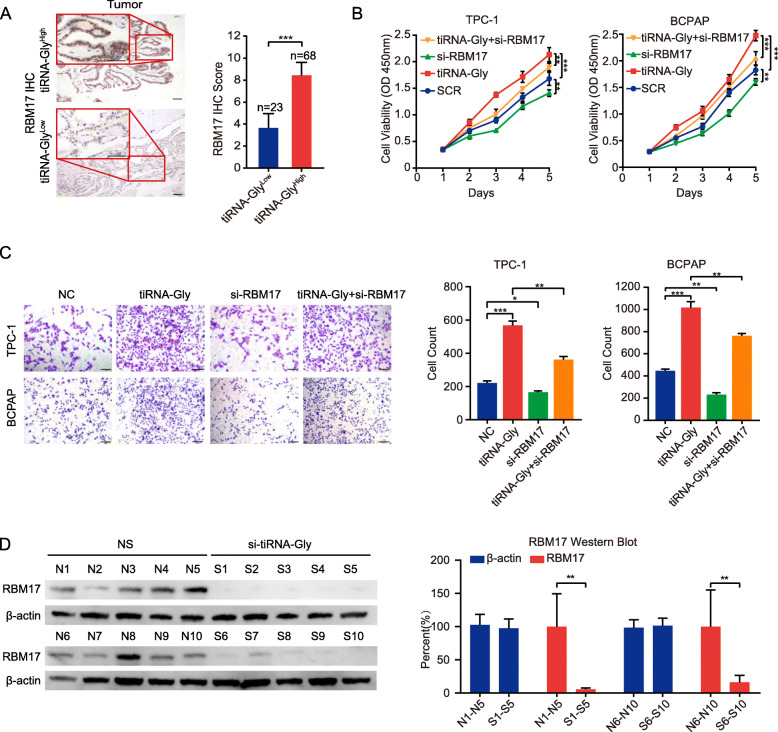
The relationship between tiRNA-Gly and RBM17 is proved. A RBM17 expression in higher tiRNA-Gly expression group (tiRNA-Gly^High^, *n* = 68) is higher than that in lower tiRNA-Gly expression group (tiRNA-Gly^Low^, *n* = 23) by IHC. Scale bar = 20 μm. **B**-**C** Rescue experiments show that tiRNA-Gly effect in CCK-8 assay and migration assay is partly compromised by RBM17 knockdown in TPC-1 and BCPAP cells. Scale bar = 100 μm. **D** RBM17 expression is downregulated in si-tiRNA-Gly group (S1–10) compared with normal saline group (NS, N1–10) in mice subcutaneous tumors by western blot and its quantification results. β-actin referred as a control. Values are triple replicated and displayed in mean ± SD. *p < 0.05, **p < 0.01, ***p < 0.001

We then examined RBM17 expression in human PTC tissue samples with high tiRNA-Gly expression levels (tiRNA-Gly^High^) or low tiRNA-Gly expression levels (tiRNA-Gly^Low^). As expected, the tiRNA-Gly^High^ group had obviously elevated RBM17 protein levels compared with the tiRNA-Gly^Low^ group (Fig. 5A). To further investigate whether tiRNA-Gly influences the proliferation and migration of PTC cells via its association with RBM17, we performed a “rescue” experiment by transfecting tiRNA-Gly with or without RBM17 knockdown. Knockdown of RBM17 significantly attenuated the effects of tiRNA-Gly on the proliferation (Fig. 5B) and migration (Fig. 5C) of TPC-1 and BCPAP cells. To compare the effect of si-tiRNA-Gly or si-RBM17 on cell proliferation, we then knockdown tiRNA-Gly (si-tiRNA-Gly) and overexpressed RBM17 (RBM17), and knockdown RBM17 again (si-RBM17). As shown in Supplementary Fig. 2D, the relative decrease caused by si-tiRNA-Gly (si-tiRNA-Gly compared with SCR) was a little bit stronger than that caused by si-RBM17 (si-tiRNA-Gly + RBM17 + si-RBM17 compared with si-tiRNA-Gly + RBM17). Data from in vivo assays also showed that RBM17 expression in the si-tiRNA-Gly group was dramatically decreased compared to that in the NS group (Fig. 5D). In summary, we hypothesized that the effects of tiRNA-Gly on the proliferation and migration of PTC cells are dependent on RBM17, the expression of which is regulated by tiRNA-Gly.

### tiRNA-Gly modulates alternative splicing of proliferation- or migration-related genes via RBM17

**Fig. 6:**
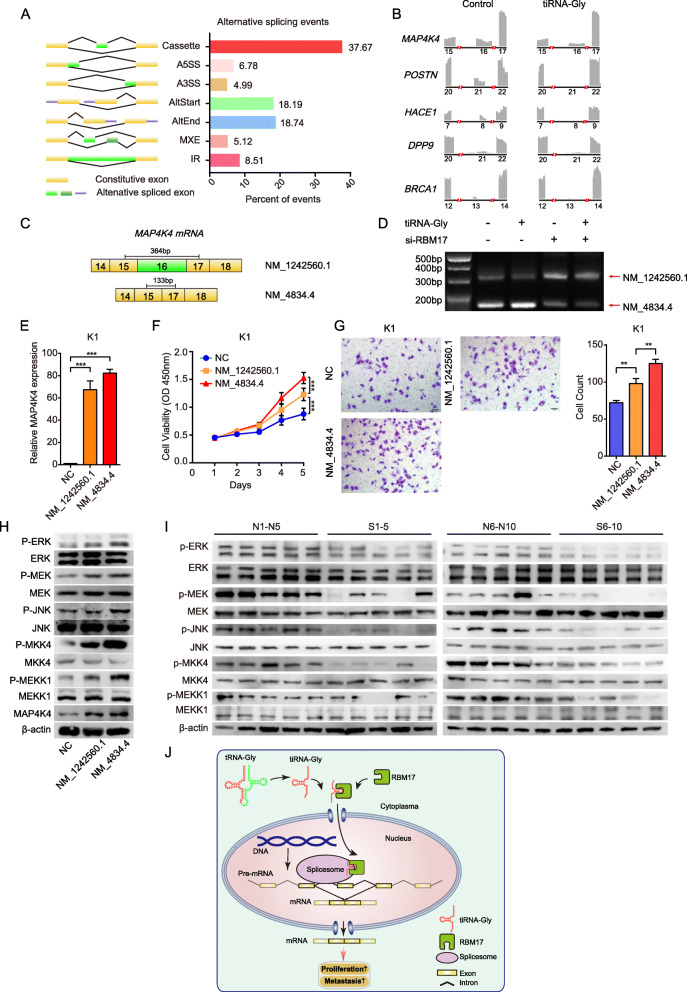
tiRNA-Gly regulates alternative splicing of *MAP4K4* mRNA via RBM17. **A** Alternative splicing induced by tiRNA-Gly is selected in K1 cells transfected with tiRNA-Gly by RNA-seq. 1622 significantly altered alternative splicing are detected, in which exons kipping (cassette) is 37.67%, alternative to 5′ splicing site (A5SS) is 6.78%, alternative to 3′ splicing site (A3SS) is 4.99%, alternative first exon (AltStart) is 18.19%, alternative end exon (AltEnd) is 18.74%, mutually exclusive events (MXE) is 5.12%, and intron retention (IR) is 8.51%. *p* < 0.05. **B** Five potential decreased alternative spliced genes were shown. Number means the location of exon. **C** The structure from exon 14 to exon 18 of long transcript NM_1242560.1 and truncated transcript NM_4834.4 of *MAP4K4* mRNA. **D** Knockdown of RBM17 blocked the increase in the truncated variant (NM_4834.4) of *MAP4K4* mRNA induced by tiRNA-Gly. **E** Expression of MAP4K4 mRNA in K1 cells transfected with two transcripts of MAP4K4 by qRT-PCR. **F**-**G** Truncated transcript NM_4834.4 has stronger effect compared with long transcript NM_1242560.1 in K1 cells in CCK-8 assay and migration assay. Scale bar = 100 μm. **H** Phosphorylation level of proteins in ERK and JNK pathways is higher in K1 cells transfected with truncated transcript NM_4834.4 compared with long transcript NM_1242560.1 by western blot. **I** The expression of total proteins and phosphorylated proteins involved in ERK and JNK pathways in xenografted cancer. Phosphorylation level of those proteins is reduced in xenografted cancer of si-tiRNA-Gly injected group (S) compared with those in normal saline group (N), while the level of total proteins does not change. **J** Working model. tiRNA-Gly binds the UHM domain of RBM17 and facilitates its translocation, giving rise to increased RBM17 protein, which induces alternative splicing of target genes in PTC cells. Values are triple replicated and displayed in mean ± SD. **p < 0.01, ***p < 0.001

Since RBM17 is a spliceosome protein that participates in the alternative splicing of mRNAs, it would be interesting to probe whether tiRNA-Gly modulates the alternative splicing of downstream genes when it binds to RBM17. RNA sequencing (RNA-seq) was thus performed in K1 cells overexpressing tiRNA-Gly. Of all the alternative splicing events shown in Fig. 6A, exon skipping (cassette exons) accounted for 37.67%, splicing at A5SSs accounted for 6.78%, splicing at A3SSs accounted for 4.99%, alternative first exon (AltStart) splicing accounted for 18.19%, alternative end exon (AltEnd) splicing accounted for 18.74%, mutually exclusive events (MXEs) accounted for 5.12%, and splicing with intron retention (IR) accounted for 8.51% of these events. Exon skipping was clearly the most frequent alternative splicing event induced by tiRNA-Gly. *MAP4K4*, *POSTN*, *HACE1, DPP9* and *BRCA1* were the genes whose expression was changed the most due to exon skipping. tiRNA-Gly induced exon 16 skipping in *MAK4K4*, exon 21 skipping in *POSTN*, exon 8 skipping in *HACE1,* exon 21 skipping in *DPP9* and exon 13 skipping in *BRCA1* (Fig. 6B). As shown in Fig. 6C-D, elevated tiRNA-Gly induced mRNA levels of a truncated variant (NM_4834.4) and decreased mRNA levels of a long variant (NM_1242560.1) of *MAP4K4*, while downregulation of RBM17 had the opposite effect. Importantly, knockdown of RBM17 blocked the increase in the truncated variant (NM_4834.4) of *MAP4K4* induced by tiRNA-Gly, indicating that tiRNA-Gly-induced alternative splicing is dependent on RBM17. Gene ontology (GO) pathway analysis showed that the following cell biological process pathways are involved in tiRNA-Gly transcription: transcription from the RNA polymerase II promoter, transcription, RNA metabolic process and the MAPKKK cascade. Gene set enrichment analysis (GSEA) revealed the top 10 most enriched oncogenic signatures in these pathways, including RAF, MTOR, JAK2 and ATF2 (supplementary Fig. 2E and 2F). Additionally, Cytoscape analysis illustrated that migration-related genes (ATP8, ND4L, IL33, ND5, FN1, CD46, INHBA) were enriched in tiRNA-Gly-overexpressing K1 cells (supplementary Fig. 2G). Thus, our results revealed that tiRNA-Gly-induced alternative splicing can alter downstream pathways and these effects are partly dependent on RBM17.

MAP4K4 is a member of the serine/threonine protein kinase family, which could activate MAPK pathway by phosphate protein MEKK1 (MAPK/ERK kinase kinase 1). To determine the effect of alternative splicing of *MAP4K4*, we constructed pCMV-NM_4834.4 and pCMV-NM_1242560.1 plasmids and respectively transfected them into K1 cells, the transfection efficiency was proved by qRT-PCR (Fig. 6E). We elucidated that both variants substantially enhanced proliferation and migration of K1 cells. Expectedly, the truncated variant (NM_4834.4) had a stronger effect on proliferation and migration than the long variant (NM_1242560.1) (Fig. 6F-G). Moreover, total MEKK1, MKK4, JNK, MEK, ERK did not altered, whereas their phosphorylation level increased in both NM_4834.4 variant and NM_1242560.1 variant transfected groups compared to control group. To be important, NM_4834.4 had a more powerful impact on phosphate-MEKK1, MKK4, JNK, MEK, ERK compared to NM_1242560.1, suggesting that the exon 16 splicing in *MAP4K4* influenced the phosphorylation of downstream proteins in MAPK pathway (Fig. 6H). The results of in vivo mouse model proved that the level of phosphorylated proteins was significantly reduced in transplanted tumors of si-tiRNA-Gly injected group, while the level of total proteins did not change compared with those in normal saline group (Fig. 6I). These results revealed that RBM17-mediated exon 16 splicing of *MAP4K4* induced by tiRNA-Gly could enhance proliferation and migration of PTC cells and phosphate downstream proteins of MAPK pathway.

## Discussion

In this study, we firstly identified a human-specific tRNA half, tiRNA-Gly was elevated in PTC tissue and found high tiRNA-Gly expression was related to more advanced clinical features in PTC patients. Functionally, tiRNA-Gly could promote cell proliferation and migration of PTC cells. Importantly, our study uncovered that tiRNA-Gly directly binded the UHM domain of splicing-related RNA-binding protein RBM17. Moreover, tiRNA-Gly facilitated RBM17 translocation and maintained its stability through ubiquitin/proteasome-dependent way. Interestingly, we revealed that tiRNA-Gly induced alternative splicing of exon 16 in *MAP4K4* mRNA and then phosphorylated MAPK pathway. Until now, little was known about tiRNA-protein interactions. Our findings demonstrate for the first time a molecular mechanism involving tiRNAs and splicing-related RNA-binding proteins and provide a novel explanation for the vital roles of tiRNAs in the tumorigenicity (Fig. 6I).

The effects of tiRNAs on different tumors are heterogeneous. Stress-induced tiRNA cleavage by angiogenin (ANG) and tRF-Leu-CAG have been reported to promote colorectal cancer metastasis [9] and the proliferation and cell cycle of lung cancer cells [7] as tumor drivers. Some tiRNAs were shown to be tumor suppressors in renal cell carcinoma [5, 6]. To be the best of our knowledge, this is the first study to identify three different tiRNAs (tiRNA-Gly, tiRNA-Lys and tiRNA-Glu) in PTC tissue. Among them, tiRNA-Gly, a 33 nt tRNA half generated from the 5′ end of pre-tRNA, was the most abundant tRNA fragment in PTC. tiRNA-Gly showed remarkably increased expression in PTC tissues and was positively correlated with the clinical-pathological characteristics of PTC. tiRNA-Gly dramatically promoted PTC cell proliferation and tumor invasion in vitro and tumorigenicity in vivo. Together, these data reveal that tiRNA-Gly may be a novel oncogenic driver in PTC and that targeting tiRNA may be a promising treatment in PTC.

tiRNAs are not well understood, and their in-depth mechanisms have not been fully illustrated. One study reported that 5′- tRNA halves displaced mRNAs binding to YB-1 and inhibited the translation stability of YB-1 to suppress the formation of stress granules [18]. A latest study found 5′-tiRNA-Val was significantly low expression in breast cancer and it suppressed Wnt/β-catenin signaling pathway through directly targeting FZD3 mRNA 3′-UTR in breast cancer cells [10]. In the current study, a fully different model in which the tiRNA-Gly directly bind to the UHM domain of splicing-related protein RBM17 was found. RBM17 is an oncogenic factor in many malignant tumors [12, 13]. Our current data proved that RBM17 was highly expressed in PTC tissues and played a strong oncogenic role by promoting proliferation and migration of PTC cells, functions similar to those of tiRNA-Gly. RBM17 has two function domains, the UHM and G-patch, which are related to RNA binding and splicing, respectively. The RBM17 UHM could bind UHM-ligand motifs (ULMs) in U2AF65 and SF1, resulting in alternative splicing of target gene [19]. As expected, we demonstrated that tiRNA-Gly could bind to the UHM domain of RBM17. tiRNAs are mainly located in the cytoplasm, but they can be imported into the nucleus by the Ran cycle [20, 21], which can transport spliceosome proteins into the nucleus as well [22, 23]. Interestingly, we observed the interaction with tiRNA-Gly gave rise to sharkly decrease in cytoplasm and obviously increase in nucleus of RBM17 in PTC cells. Thus, we assumed that tiRNA-Gly was cleaved from tRNA-Gly and interacted with RBM17 UHM in the cytoplasm, after which Ran cycle translocated the tiRNA-Gly/RBM17 complex into the nucleus of PTC cells. RBM17 then accumulated in the nuclei partly because tiRNA-Gly stabilized the RBM17 protein in the way of ubiquitin/proteasome-dependent degradation. Notably, our study enriches understanding of the molecular mechanism by which tiRNAs in tumor cells bind protein partners and act at the posttranscriptional level.

The lncRNA Saf has been reported to interact with RBM17 to induce exon 6 splicing of the *Fas* gene, a novel mechanism by which a non-coding RNA modulates the cell death program [14]. To elucidate whether the tiRNA-Gly can mediate the alternative splicing of target genes via its interaction with RBM17, we performed RNA-seq in tiRNA-Gly-overexpression PTC cells. The data showed that at least 500 gene isoforms were spliced, and among splicing events, exon skipping was the most frequent. Importantly, we proved that the exon 16 skipping of *MAP4K4* gene induced by tiRNA-Gly was RBM17-dependent. MAP4K4 is a serine and threonine protein kinase that belongs to the STE20/MAP4K family [24]. It could promote hepatocellular carcinoma growth and migration of lung adenocarcinoma cells through MAPK pathway [25–27]. Previous study reported that RBM4a-SRSF3 modulated alternative splicing of *MAP4K4* and various transcript variants of *MAP4K4* acted slightly different on JNK pathway [28, 29]. Herein, we found that tiRNA-Gly/RBM17 complex induced two distinct isoforms of *MAP4K4* (NM_4834.4 and NM_1242560.1). Interestingly, our present study showed that the truncated isoform (NM_4834.4) exhibited more promoting effect than the long isoform (NM_1242560.1) in proliferation and migration of PTC cells, and activated more phosphorylation of downstream proteins in MAPK pathway. Such data revealed that the alternative splicing induced by tiRNA-Gly via its binding with RBM17 UHM leaded to activation of oncogenic signaling pathway in PTC cells. Moreover, it has been reported that RBM17 is the substrate for ERK, JNK and p38 MAP kinase. They could enhance the function of RBM17 by regulating its splice site utilization and phosphorylation [13]. We presumed that it may make a positive feedback in tiRNA-Gly/RBM17/MAP4K4 axis and may accelerate the tumor-promoting role of tiRNA-Gly in PTC. Therefore, we hypothesized that tiRNA-Gly displayed a strong tumor-promoting effect on PTC through inducing alternative splicing via the tiRNA-Gly-RBM17 interaction.

## Conclusions

In summary, we revealed that a tiRNA-Gly acts as a tumor oncogene in PTC. tiRNA-Gly can bind the UHM of RBM17, resulting in the translocation and upregulation of RBM17. tiRNA-Gly exerts its oncogenic effect by inducing RBM17-dependent alternative splicing. These findings regarding tiRNA-Gly provide novel insight into the molecular interaction between tRNA fragments and RNA-binding proteins and may be helpful in developing precise approaches for tumor screening and treatment.

## Supplementary Information

**Additional file 1.**

## Data Availability

The data in the current study are available from the corresponding authors on reasonable request.

## Abbreviations

sncRNAs: Small non-coding RNAs
tiRNA: tRNA halves
tRFs: tRNA derived fragments
PTC: Papillary thyroid carcinoma
lncRNA: Long noncoding RNAs
UTR: Untranslated region
RBM17: RNA binding motif protein 17
UHM: U2AF homology motif
RT-PCR: Reverse Transcription PCR
IP: Immunoprecipitations
CCK-8: Cell counting kit-8
RIP: RNA immunoprecipitation
IF: Immunofluorescence
IHC: Immunohistochemistry
H&E: Hematoxylin and eosin
T: Tumor tissue
AT: Adjacent tissue
tiRNA-Gly: 5′-tiRNA-Gly-GCC
NS: Normal saline
CHX: cycloheximide
RNA-seq: RNA sequencing
AltStart: Alternative first exon
AltEnd: Alternative end exon
MXE: Mutually exclusive event
IR: Intron retention
GO: Gene ontology
GSEA: Gene set enrichment analysis
ANG: Angiogenin
ULM: UHM-ligand motifs
