# Supplementary material for: A 5′-tRNA halve, tiRNA-Gly promotes cell proliferation and migration via binding to RBM17 and inducing alternative splicing in papillary thyroid cancer
Source: J Exp Clin Cancer Res. 2021 Jul 5;40:222. doi: 10.1186/s13046-021-02024-3 (PMC8256553; doi:10.1186/s13046-021-02024-3)
Supplement: Supplementary file 1 — Additional file 1. [file 13046_2021_2024_MOESM1_ESM.docx]

**Supplementary Table 1. Clinical characteristics of 91 patients**

| **Clinicopathologic parameters** | tiRNA-Gly expression | | ***p*-value** |
| --- | --- | --- | --- |
|  | High (%) | Low (%) |  |
| **Gender** |  |  | 0.894 |
| Male | 15(51.7) | 14(48.3) |  |
| Female | 33(53.2) | 29(46.8) |  |
| **Age (years)** |  |  | 0.492 |
| < 55 | 28(56.0) | 22(44.0) |  |
| ≥ 55 | 20(48.8) | 21(51.2) |  |
| **Tumor size (cm)** |  |  | 0.000^***^ |
| ≤ 1 | 10(27) | 27(73.0) |  |
| > 1 | 38(70.4) | 16(29.6) |  |
| **Extrathyroidal invasion** |  |  | 0.579 |
| Positive | 24(50.0) | 24(50.0) |  |
| Negative | 24(55.8) | 19(44.2) |  |
| **Lymph node metastasis** |  |  | 0.000^***^ |
| Positive | 42(70.0) | 18(30.0) |  |
| Negative | 6(19.4) | 25(25) |  |
| **TNM stage 8^th^** |  |  | 0.005^**^ |
| I, II | 40(48.2) | 43(51.8) |  |
| III, IV | 8 (100.0) | 0(0.0) |  |
| **Bilateral location** |  |  | 0.024^*^ |
| Positive | 22(68.8) | 10(31.3) |  |
| Negative | 26(44.1) | 33(55.9) |  |

**p*< 0.05; ****p*< 0.001

**Supplementary Table 2. Mass spectrometry result**

| **Gene Name** | **PepCount** | **Unique**  **PepCount** | **Cover**  **Percent%** | **MW** | **PI** |
| --- | --- | --- | --- | --- | --- |
| RBM17 | 7 | 6 | 10.72% | 44961.01 | 5.76 |
| DCD | 4 | 4 | 22.73% | 11283.74 | 6.09 |
| HIST1H1E | 3 | 3 | 12.33% | 21893.04 | 11.03 |
| HIST1H1D | 3 | 3 | 12.22% | 22349.61 | 11.02 |
| HIST1H1C | 3 | 3 | 12.68% | 21364.47 | 10.94 |

MW: Molecular Weight

**Supplementary Figure 1.**

**A** Template diagram of primer for tiRNA-Gly used in qRT-PCR.

**B** Fold change of tiRNA-Gly copy number variations in tumor are more than adjacent tissues in 91 pairs of human PTC.

**C** Structure of two tiRNA-Gly fragements (1297, 1293) and total tRNA-Gly.

**D** Higher tiRNA-Gly expression is positively correlated with tumor size, lymph nodes metastasis and advanced stage of PTC.

**E** Template diagram of siRNAs designed for tiRNA-Gly (si-tiRNA-Gly).

**F** The effect of si-tiRNA-Gly 2-4 on total tRNA-Gly. SCR acts as wild type.

**G** The effect of si-tiRNA-Gly 2-4 on tiRNA-Gly. SCR acts as wild type.

**H** The effect of si-tiRNA-Gly 2-4 on cell viability of TPC-1 and BCPAP cells. SCR acts as wild type.

**I** tiRNA-Gly in transplanted tumors of si-tiRNA-Gly group (S) was significantly reduced compared with those in normal saline group (N).

**Supplementary Figure 2.**

**A** RIP assays confirm the binding between RBM17 and tiRNA-Gly in K1 cells. Rabbit IgG acts as negative control, 10% input acts as positive control. Anti-FLAG antibody used in RIP assay.

**B** Quantification results of cell fraction when tiRNA-Gly overexpression facilitates RBM17 translocation from cytoplasm to nucleus by western blot. GAPDH acts as cytoplasm control and Histone H3 as nucleus control.

**C** Quantification results of upregulated RBM17 expression after tiRNA-Gly transfection in K1 cells by western blot.

**D** The effect of si-tiRNA-Gly and/or si-RBM17 on cell proliferation in TPC-1 and BCPAP cells.

**E** Gene set enrichment analysis shows enrichment of GO biological process related pathway in K1 cells transfected with tiRNA-Gly.

**F** Gene set enrichment analysis shows enrichment of genes in cancer-related pathways in K1 cells transfected with tiRNA-Gly.

**G** Genes upregulated in tiRNA-Gly transfected K1 cells are enriched in Cytoscape software. Blue dots mark enrichment genes related with migration.
